# Cancer cell metabolic reprogramming: a keystone for the response to immunotherapy

**DOI:** 10.1038/s41419-020-03175-5

**Published:** 2020-11-11

**Authors:** Michaël Cerezo, Stéphane Rocchi

**Affiliations:** 1 INSERM U981, Gustave Roussy, Villejuif, France; 2 INSERM U1065, Team 12, Centre Méditerranéen de Médecine Moléculaire, Université Côte d’Azur, Nice, France; 3 Present Address: INSERM U1065, Team 12, Centre Méditerranéen de Médecine Moléculaire, Université Côte d’Azur, Nice, France

**Keywords:** Cancer metabolism, Immunotherapy

## Abstract

By targeting the tumor microenvironment to stimulate antitumor immunity, immunotherapies have revolutionized cancer treatment. However, many patients do not respond initially or develop secondary resistance. Based on the limited resources in the tumor microenvironment and competition between tumor and immune cells, the field of immune metabolism has produced extensive knowledge showing that targeting metabolism could help to modulate antitumor immunity. However, among all the different potentially targetable metabolic pathways, it remains unclear which have more potential to overcome resistance to immune checkpoint inhibitors. Here, we explore metabolic reprogramming in cancer cells, which might inhibit antitumor immunity, and strategies that can be used to favor the antitumor response.

## Facts

- Immunotherapy targeting the tumor microenvironment (TME) has changed the paradigm of cancer treatment.
- Cancer cells and antitumor effector cells share metabolic dependencies.
- Targeting cancer cell metabolism could be key to bypassing immune checkpoint blockade (ICB) resistance

## Open questions

- Which pathway(s) target to favor anti-tumor immune response?
- How monitored metabolic competition between cancer and immune cells in patients to determine when is time to interfere?

## Introduction

During the last decade, the paradigm of cancer therapy has been revolutionized by the development of immunotherapies. Indeed, for the first time, the goal is to stimulate the host immune system to attack tumor cells, while previous treatments were designed to directly target cancer cells. Historically, after the proof of concept^1^, the first evidence of the efficiency of immunotherapy was in the treatment of cutaneous melanoma with antibodies targeting cytotoxic T lymphocyte-associated protein 4 (CTLA-4). CTLA4 is a negative checkpoint protein expressed at the plasma membrane of resting T cells after T cell receptor (TCR) engagement and costimulatory signaling through CD28. CTLA4 competes with CD28 for the binding of CD80 and CD86 and induces the inhibition of T cell activation. However, even if some durable responses were observed to antibodies targeting CTLA4 (ipilizumab) in patients with metastatic melanoma, the overall response was modest and unfortunately associated with frequent toxicities resulting from tissue-specific inflammation^2,3^.

Then, a second strategy was developed based on the targeting of the interaction between PD1 and PD-L1^4^. The overexpression of PD-L1 is an adaptive resistance mechanism, which tumor cells utilize to escape the antitumor immune response via PD-1–mediated T cell exhaustion. The first evidence of the antitumor activity of antibodies targeting PD1 (e.g., nivolumab and pembrolizumab) was obtained in patients with melanoma and non-small cell lung cancer (NSCLC). Interestingly, adverse events are less frequent for anti-PD1 treatment than for anti-CTLA4 treatment, and durable responses are observed in ~30% of patients treated with anti-PD1^5,6^. Currently, there are five anti–PD-1 or anti-PD-L1 antibodies approved by regulatory agencies for the treatment of 11 different cancer types^7^. Finally, a combination strategy was developed utilizing a combination of anti-CTLA4 and anti-PD1 antibodies. However, even if the efficiency is greatly enhanced, unfortunately, the frequency and intensity of adverse events increase dramatically, limiting the utilization of this combination^8^.

It seems that cancer treatment with immunotherapy has now reached a plateau, which cannot be crossed without a better understanding of the adaptive mechanisms underlying the resistance of cancer cells to treatment.

Cancer cells are characterized by their plasticity. Indeed, cancer cells will alter many different cellular processes to adapt to stress conditions and to continue to proliferate. Concerning the specifics of cell metabolism, since the 1920s, it has been known that metabolic reprogramming is a hallmark of transformation^9^. However, due to the important needs for energy and building blocks, we know now that the reality of metabolic reprogramming in cancer cells is infinitely more complex than that implied by this first observation^10^.

Due to the consumption of resources by cancer cells and vascularization impairments, the tumor microenvironment is frequently poor in nutrients and oxygen, establishing competition between cancer and stromal cells. Because immune checkpoint therapies target immune effector cells and do not directly target cancer cells, metabolic crosstalk between these two cell populations appears to be a determinant of the effects of immunotherapy. Here, we have decided to focus our interest on some metabolic adaptations in cancer cells that can interfere directly or indirectly with T cell effector and immune checkpoint blockade efficiency.

## Tumor cells starve T cells to block antitumor immunity

Even though numerous studies have shown that oxidative phosphorylation (OXPHOS) is intact in many different tumors, the first described metabolic characteristic of cancer cells was their preference for conversion to the production of ATP through anaerobic glycolysis^11^ to adapt to hypoxic conditions prevailing during tumor development^12^. The high demand for glucose of cancer cells creates competition in the tumor microenvironment that has a negative impact on neighboring cells, such as immune cells^13,14^. Interestingly, during their activation, proliferation, and differentiation, T cells alter their metabolism. In the quiescent state, naïve T cells rely mainly on OXPHOS and fatty acid oxidation (FAO) to support their needs. After activation through the T cell receptor (TCR) and costimulatory receptor engagement, T cells alter their metabolism to support proliferation and effector functions^15^. In particular, CD28 costimulatory engagement activates the PI3K/AKT pathway and increases glycolytic flux^16,17^. The tremendous increase in glycolysis flux supports the pentose phosphate pathway (PPP), serine biosynthesis, and fatty acid synthesis pathway and produces intermediates for nucleotide synthesis^18^. Thus, because the availability of glucose in the tumor microenvironment is limited, competition between cancer cells and T cells for glucose appears to be a potential key determinant of whether the overall antitumor immune response will lead to tumor elimination or growth and resistance to antitumoral immune surveillance. In support of this idea, melanoma cells isolated from patients with high levels of glycolysis showed a reduced response to adoptive T cell therapy^19^. However, recently a study has shown that a transitory glucose restriction can enhance CD8 T cell effector functions highlighting the complexity of the crosstalk between metabolic and effector functions^20^.

As amino acids are protein building blocks, the high availability of amino acids is essential for tumor growth. However, amino acids are also required by immune cells to differentiate and develop their effector functions and ultimately control tumor development. Considering this, a better understanding of the utilization of amino acids by each population of cells in the tumor microenvironment appears essential to be able to stimulate antitumor immunity efficiently.

Glutamine, through glutaminolysis, fuels the tricarboxylic acid (TCA) cycle to provide metabolic intermediates that serve as building blocks for lipids, proteins, and nucleic acids, which are necessary for cancer cell proliferation^21,22^. Interestingly, this same metabolic pathway has been shown to be essential for T cell activation and proliferation^18,23^. More specifically, during T cell activation, the MAPK/ERK pathway coordinates the upregulation of glutamine uptake and glutaminolysis^23^. Due to the activation of oncogenic signaling pathways, such as the MAPK/ERK pathway, cancer cells increase their glutamine uptake and utilization^24^. Thus, competition for glutamine between cancer cells and activated T cells can have a negative impact on T cell and antitumor immune responses, but it can also present therapeutic opportunities. Even though targeting glutamine metabolism has shown a modest effect globally in vivo^25,26^, Leone et al. have recently shown that glutamine metabolism inhibition using an analog of the broad spectrum inhibitor 6-diazo-5-oxo-L-norleucine (DON) can enhance immune checkpoint inhibitor efficiency due to glutaminolysis blockade, which can be compensated for in CD8^+^ T cells but not in cancer cells^27^. To survive DON treatment, cells need to adapt their metabolic flux. However, cancer cells, that accumulate metabolic vulnerabilities, appear less flexible from a metabolic point of view compare to CD8 + T cells. Even if further studies will be necessary to fully understand the dichotomic effect in cancer cells and CD8 + T cells, we can speculate that which could explain the anti-tumor immune response observed.

Glutamine blockade can also have an indirect immunostimulatory antitumor effect, as illustrated by the fact that a small-molecule inhibitor of glutamine metabolism inhibited the generation and recruitment of myeloid-derived suppressor cells (MDSCs) via the inhibition of Colony Stimulating Factor 3 (CSF3) production^28^. Interestingly, in this study, the authors showed that glutamine metabolism inhibition also has an effect on tryptophan catabolism via IDO suppression. However, Ma et al. demonstrated that in renal cancer, glutamine deprivation induced the expression of PD-L1^29^, showing the difficulty of targeting glutamine in the context of immunotherapy.

Tryptophan is an essential amino acid that cells utilize through the kynurenine pathway^30^. Because tumor cancer cells and T cells need to import tryptophan from the microenvironment for their needs, there is competition for this resource. Specifically, in T cells, tryptophan availability is critical for proliferation^31^. After stimulation, T cells upregulate the expression of several amino acid transporters, including SLC7A5. Interestingly, SLC7A5 knockdown blocks T cell clonal expansion and effector differentiation by activating mTOR and inducing the expression of c-Myc^32^. Thus, tryptophan catabolism is an important mechanism by which cancer cells can inhibit the antitumor immune response. The most studied pathway of tryptophan catabolism involves indoleamine-2,3-dioxygenase (IDO), an enzyme that catalyzes the conversion of tryptophan to kynurenine. Interestingly, IDO expression can be stimulated by type I interferon (interferon-αβ) and type II interferon (interferon-γ)^33^ because IDO1 gene promoters possess IFN-stimulated response elements (ISREs) and IFN-activated sites (GAS). Interferon-γ is one of the hallmarks of activated CD8 + T cells, and IDO expression, like PD-L1 expression, by cancer cells can be seen as an adaptive mechanism that cells adopt in response to CD8 + T cell infiltration in tumors. Two other enzymes have been shown to be IDO-related enzymes, indoleamine-2,3-dioxygenase 2 (IDO2) and tryptophan-2,3-dioxygenase (TDO), but they show different patterns of expression^34–36^. TDO seems to be more interesting in the cancer context, since it has been shown to be able to sustain tryptophan catabolism and induce inhibition of the antitumor immune response^37,38^.

Tryptophan starvation inhibits CD8 + T cell effector functions and stimulates CD4 + regulatory T (Treg) cell functions, creating robust immunosuppression that can affect immune checkpoints mediated by the CTLA4 and PD1/PD-L1 pathways to create a tolerogenic tumor microenvironment. Mechanistically, the effects are mediated through the activation of the stress response kinase GCN2, which inhibits mTORC2 and downstream AKT^39,40^. Initially, it was proposed that IDO mediated the immunosuppressive effect directly through local tryptophan starvation^41^. In support of these findings, IDO1 expression has been associated with poor prognosis in multiple tumor types^42,43^. However, it has been shown in B16 melanoma model studies that the suppression of the antitumor T cell response induced by tryptophan catabolism can be mediated independently of the effect of GCN2^44^.

Interestingly, numerous studies have shown that metabolites generated through tryptophan catabolism, such as kynurenine, kynurenic acid, 3-hydroxy-kynurenine, and 3-hydroxy-anthranilic acid, can suppress T cell-mediated antitumor immunity^45^. Kynurenine, the first metabolite product in the IDO-dependent tryptophan degradation pathway, can be exported to the tumor microenvironment by cancer cells to inhibit antitumor immunity and prevent tumor clearance^37,46,47^. Several studies have identified that kynurenine can activate the aryl hydrocarbon receptor (AhR)^37,48^. Functionally, AhR is a cytosolic protein that will translocate to the nucleus after interaction with its ligands. In the nucleus, AhR binds the promoter regions of target genes containing sequences called “aryl hydrocarbon response elements” (AHREs) as well as dioxin-response elements (DREs)^49–51^. Interestingly, AhR expression is enriched in interleukin 17 (IL-17)-producing CD4 + T cells and is implicated in the generation of regulatory T cells (Tregs)^52–54^. Another study has also shown that the immunosuppressive effect of AhR after its interaction with kynurenine can be mediated by the alteration of CD8 + T-cell function^37^.

The interconnection between the inhibition of antitumor immunity mediated via GCN2 activation that is induced by tryptophan depletion and kynurenine-mediated AhR translocation needs to be studied more deeply, but one study has already shown that these two pathways can cooperate to induce a regulatory T cell phenotype and permit tumor development^55^. One possible connection between these two mechanisms could be the amino-acid transporter LAT1, which has been shown to import tryptophan into the cell and, at the same time, to function as an antiport system for kynurenine^56^.

Altogether, the evidence suggests that tryptophan catabolism appears to be one of the major metabolic mechanisms driving the inhibition of the antitumor immune response and is therefore a major therapeutic target that we will discuss later in this review.

Arginine is another amino acid that has limited availability in the tumor microenvironment. A recent study showed that amino acids are the most depleted substances in tumors^57^. Due to the common need for arginine of tumor cells and immune cells, arginine starvation in immune cells appears to be another road used by cancer cells. Indeed, L-arginine uptake has been shown to be necessary for CD8^+^ T cell proliferation, memory response formation, and finally antitumor responses^58^. The absence of arginine availability has been shown to interfere with glycolysis in T cells, leading to the inhibition of cytokine production and T cell proliferation^59,60^.

However, the case of arginine is particularly interesting because even if some studies have shown that cancer cells produce arginases, which comprise the enzyme family responsible for arginine degradation^61–63^, a large proportion of the literature shows that the majority of arginases come from the tumor stroma. Indeed, it is principally the MDSCs, whose accumulation in the tumor stroma is a hallmark of tumor development, that are responsible for arginine deprivation. Indeed, during its development, the tumor corrupts the myeloid compartment, leading to the development of MDSCs^64^ that express arginases and inhibit antitumor immunity^65,66^.

**Fig. 1:**
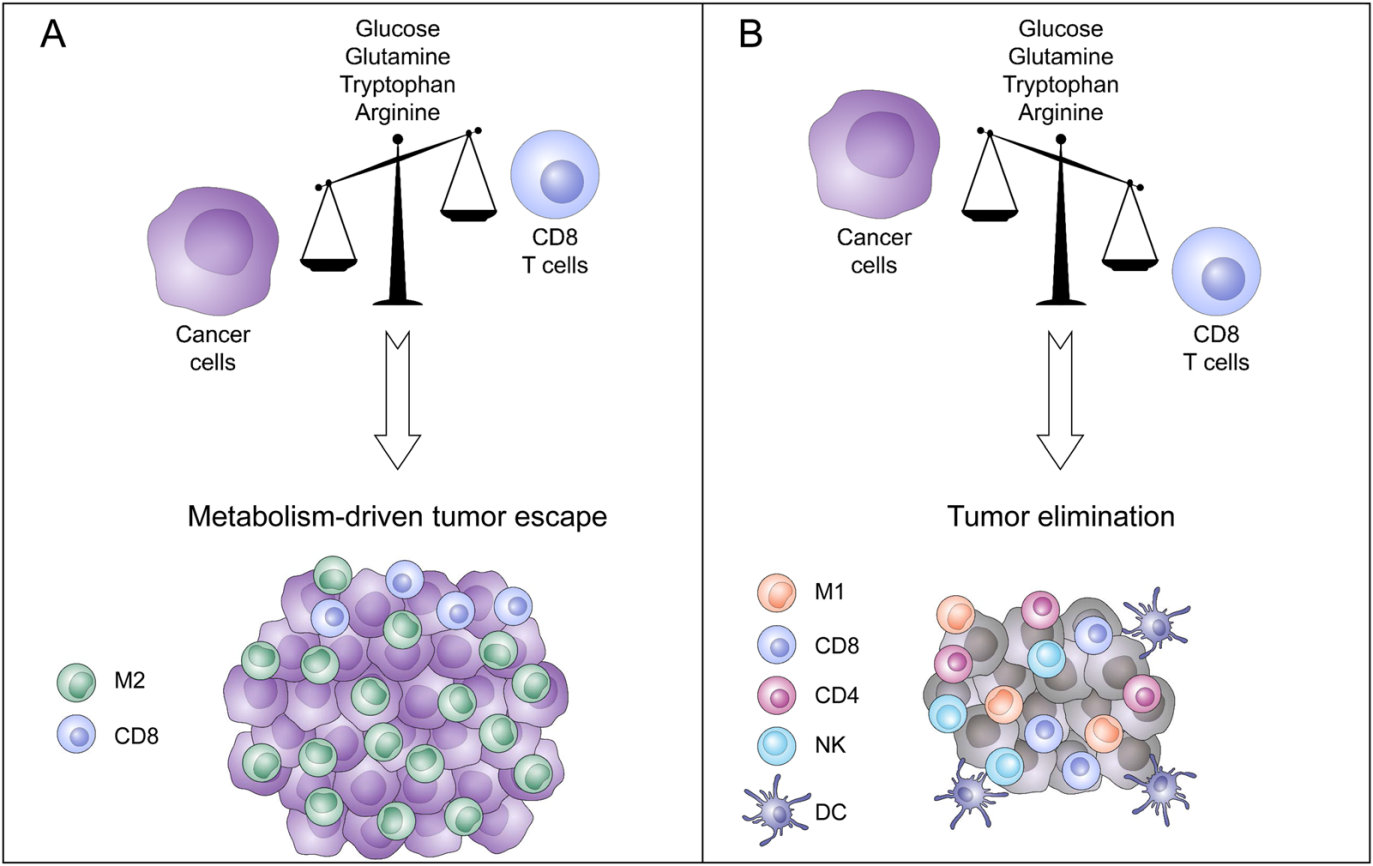
Metabolic competition in the micro-environment determines tumor outcome. Tumors cells and effector T cells share dependencies for glucose, glutamine, tryptophan, or arginine. **a** Tumor cells, by their extensive need of glucose, glutamine, tryptophan of arginine will impoverish the tumor area. This resource consumption from tumor cells will directly impact CD8 T cell metabolism leading to exhausted phenotype and indirectly, through metabolite secretion like for example lactate, stimulate the development of an immunosuppressive tumor micro-environment. **b** At the opposite, if tumor cells have moderate metabolic demand, due to intrinsic characteristics or therapeutic manipulations, a fully functional anti-tumor immune response will mediated tumor elimination.

Taken together, these studies highlight the importance of arginine availability for antitumor immunity efficiency and point to potential actionable mechanisms that could be used to target MDSC-dependent antitumor immunity suppression (Fig. 1).

## Tumor cell metabolism creates a hostile tumor microenvironment

Interestingly, not only the limitation of glucose availability for T cells but also the increase in glycolysis flux, which will produce immunosuppressive metabolites such as lactate, can reduce tumor immune rejection and responses to immunotherapy. Indeed, lactate is produced from pyruvate by the glycolytic enzyme LDH. Interestingly, a high LDH level is associated with poor clinical outcomes for various tumor types^67^. Even if it is commonly admitted that the level of LDH is a reflection of the tumoral mass, the reason for the predictive value of LDH remains controversial. The accumulation of lactate in the tumor microenvironment is associated with acidification of the tumor area. Indeed, monocarboxylate transporters (MCT1 and 4) cotransport lactate and protons from the cytoplasm to the extracellular space^68,69^. This production of lactate in tumor cells impacts interferon-gamma production by tumor-infiltrating T cells, NK activation, and the proportion of myeloid-derived suppressor cells. This will result in decreased immune surveillance and thus support tumor growth^70,71^. Lactate can also impact dendritic cells by modulating their differentiation. Indeed, lactate promotes a tumor-associated dendritic cell phenotype characterized by a decrease in major histocompatibility complex I molecules and the maintenance of a tolerogenic phenotype^72,73^. Concerning the effect of lactic acid on macrophages, the results of different studies are more contradictory, even though Colegio et al. have shown that a high concentration of lactate promotes M2 macrophage polarization through the stabilization of HIF1α^74^. Recently, lactate has been also implicated in lactate-derived lactylation of histone lysine residues. This epigenetic modification stimulates gene transcription of M2 marker genes, such as IL6 and ARG1^75^.

Apart from the direct effects of lactate, the fact that the export of lactate from cancer cells induces a pH decrease in the tumor microenvironment will also impact immune cells^76^. For example, pH decreases impair T cell cytotoxicity and the natural cytotoxicity of NK cells^77–79^. Acidosis induced by tumors can also promote macrophage polarization toward a tumor-associated macrophage phenotype that will sustain tumor growth^80^. More specifically, in the context of immunotherapy, Bosticardo et al. have shown that in addition to interfering with activation (via interleukin-2 and interferon-gamma secretion) and proliferation, a low pH also upregulates CTLA-4 expression on T lymphocytes^81^. Mechanistically, Pilon-Thomas et al. suggested that the inhibition of the immunotherapy effects mediated by tumor could be mediated by specific acid-sensing receptor(s)^82,83^, such as G-proteins, T-cell inhibitory receptor and T-cell death–associated gene-8, which have been shown to suppress MYC translation in lymphocytes^84,85^.

**Fig. 2:**
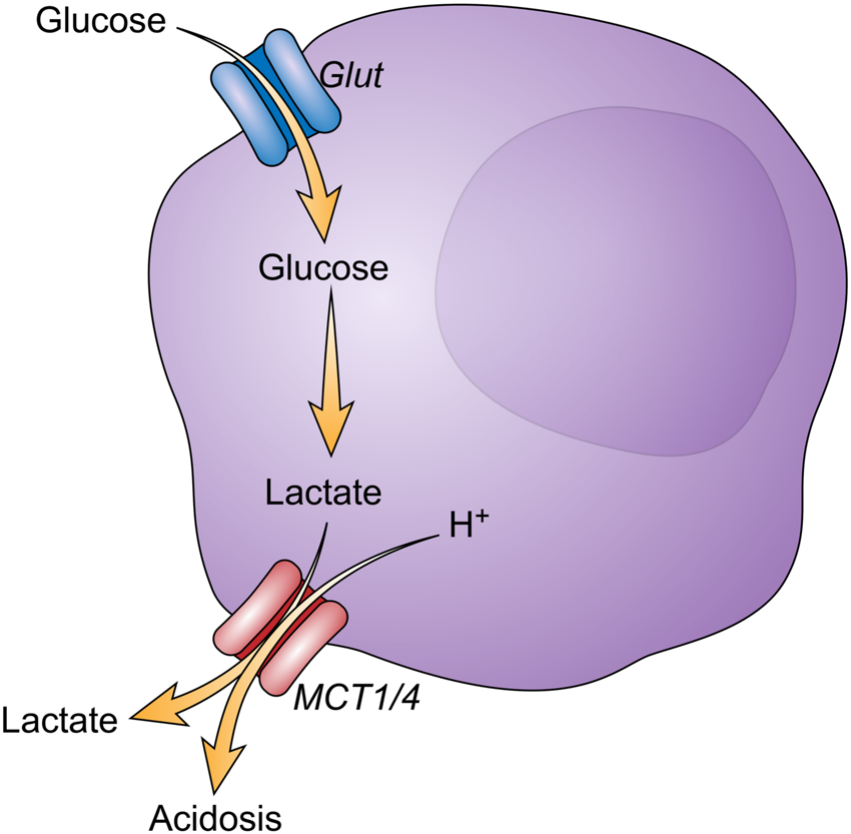
Tumor cell glycolysis creates an acidic micro-environment. Tumor cells, through aerobic glycolysis, produce lactate that will be exported in the tumor micro-environment via MCT1 and MCT4 transporters. Lactate has a direct tolerogenic effect by modulating T cells and NK cells activation as well as tumor-associated dendritic cells differentiation and M2 macrophages polarization. Lactate export also impacts micro-environment pH by inducing acidosis that will participate in the impairment of T cells and NK cells cytotoxicity, M2 polarization, and upregulation of immune checkpoints.

Altogether, these studies highlight the fact that tumor cells, apart from their direct starvation effect, can use glycolysis and related processes to model a hostile tumor microenvironment to escape antitumor immunity and continue to proliferate (Fig. 2).

## Targeting tumor cell metabolism increases immune checkpoint blockade efficiency

There is an extensive number of studies that have tried to target different metabolic pathways to increase immune checkpoint blockade efficiency or bypass resistance. However, cancer cells and immune cells from the tumor stroma, especially activated CD8 + T cells, share metabolic dependencies, making it difficult to obtain combinatory effects with drugs targeting metabolic processes and immune checkpoint blockade.

A perfect illustration of this complexity can be highlighted by the targeting of fatty acid metabolism. Indeed, even if lipid and cholesterol accumulation has been shown to correlate with cancer aggressiveness^86^, opening therapeutic opportunities especially via targets such as fatty acid synthase (FASN)^87^, the consequences on the different immune cell populations can be opposing. For example, fatty acid oxidation (FAO) is essential for the development of CD8 + memory cells^88^ as well as the function of immunosuppressive regulator T cells (Treg)^89^ and immunosuppressive M2 macrophages^90^.

Because resources are limited in the tumor microenvironment, cells are subjected to energetic stress. AMP-activated protein kinase (AMPK) is a serine/threonine kinase that functions to sense energy homeostasis through detecting the AMP/ATP ratio^91^. Even if AMPK activation has been shown to modulate various functions of cells composing the tumor stroma, including MDSCs and T cells, we have decided to focus our interest on the consequences of AMPK activation in tumor cells, which can impact the antitumor immune response. Targeting AMPK has been extensively demonstrated to be a potential antitumor strategy. The most well-characterized molecule that activates AMPK is probably the anti-diabetic drug metformin^92^. Extensive literature exists on the potential anti-neoplasic effect of metformin on various tumor types through multiple mechanisms. Concerning antitumor immunity, the first evidence was published by Eikawa et al. in 2015. The authors have shown that metformin treatment stimulates CD8 + T cell effector functions in an AMPK-dependent manner, leading to tumor regression^93^. These results have been confirmed by the fact that targeting mitochondrial oxidative phosphorylation (which is also targeted by metformin) synergizes with PD-1 blockade by inducing the expansion of cytotoxic T lymphocyte effector/memory cells in tumors and the draining lymph node due to the activation of AMPK, mammalian Target Of Rapamycin (mTOR), PPAR-gamma coactivator 1α (PGC1α) and T-bet^94^.

However, the impact of AMPK modulation on cancer cells is controversial.

On the one hand, the expression of liver kinase B1 (LKB1), one of the kinases that phosphorylates AMPK, has been shown to be correlated with PD-L1 expression in non-small cell lung cancer. Mechanistically, LKB1 increases PD-L1 expression via AMPK and KEAP1/NRF2 signaling^95^. Moreover, LKB1 knockdown was demonstrated in the same study to control cytokine production via a decrease of CCL5 and CXCL12 (chemokines recruiting lymphocytes and dendritic cells) and increases of CXCL5 and CXCL7 (chemokines promoting the recruitment of neutrophils), leading to the reshaping of the tumor microenvironment towards immunosuppressive stroma.

On the other hand, activation of AMPK by metformin has been shown to induce the phosphorylation of serine 195 of PD-L1, leading to abnormal glycosylation and finally the degradation of PD-L1 through ER-associated protein degradation (ERAD)^96^. Thus, targeting AMPK using metformin could be an alternative strategy used in combination with anti-CTLA4 therapy to stimulate antitumor immunity. However, depending on the context, the fact that AMPK activation seems to be able to increase the expression or induce the degradation of PD-L1 is intriguing, and additional studies will be necessary prior to considering a clinical use of metformin or other AMPK activators in the context of immunotherapies.

**Fig. 3:**
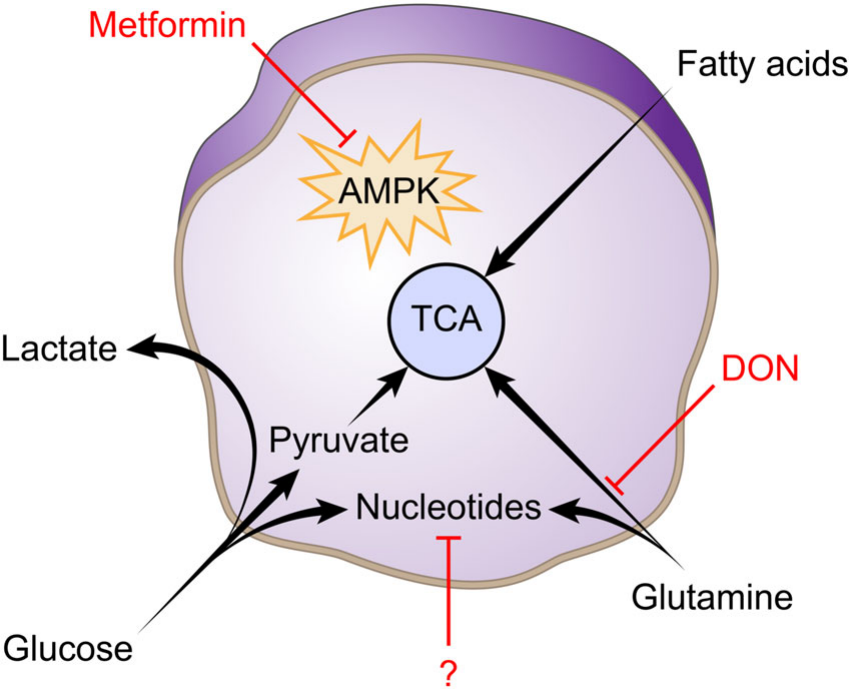
Potential actionable pathways in cancer cells to increase immune checkpoints blockade efficiency. Highly resources demand cancer cells necessary to sustain their proliferation opens several therapeutics opportunities to improve immune checkpoints blockade efficiency. Targeting AMPK especially with metformin of other biguanide derivatives could be an interesting combinatory strategy to increase immunotherapies efficiency. However, regarding the contradictory effects report on PD-L1 expression and degradation, additional exploration needs to fully evaluate the potential of this combination and especially with which immunotherapy (anti-PD1/PD-L1 or anti-CTLA4) targeting AMPK could be a therapeutic option. Glutamine dependency is share between a cancer cell and CD8 effector T cells highlighting glutaminolysis as a potential therapeutic target. Even if using single enzyme inhibitors have shown a modest therapeutic effect, recent results obtained with DON, a glutamine analog that inhibits a large spectrum of glutaminolysis enzyme have shown a spectacular effect by inducing a glutaminolysis that cannot be compensated by cancer cells contrariwise of CD8 T cells that increase glucose uptake to fuel PPP activity and maintain their anti-tumor activity. Nucleotides metabolism emerges as a promising therapeutic option especially with recent studies implicating nucleotide imbalance in the generation of a specific mutation pattern that predicts response to immunotherapy. However, exploration is needed to explore the consequences of nucleotides metabolism intervention, especially in the tumor stroma.

To support the requirements of proliferation, cancer cells increase nucleotide metabolism to provide the necessary pool of nucleotides for nucleic acid and protein synthesis. Thus, nucleotide metabolism plays an essential role in cancer cell biology and constitutes a potential target for the improvement of cancer therapy. Strategies targeting nucleotide metabolism were developed a long time ago, such as the use of antimetabolites, which competitively inhibit the activity of enzymes involved in nucleotide synthesis^97,98^. Even if these strategies are currently used for the therapy of numerous cancer types or if emerging strategies specifically targeting purines or pyrimidines appear attractive^99^, there are relatively low amounts of data concerning the potential of targeting purine or pyrimidine metabolism in the context of immunotherapy. However, recent studies have shown that alteration of the urea cycle, which is the main pathway used by mammals to eliminate waste nitrogen, can modulate the response to immunotherapy^100,101^. Indeed, authors have demonstrated that specific alterations in the expression of urea cycle-associated enzymes induce a specific mutation signature due to an increase in the ratio of pyrimidine to purine associated with the expression of hydrophobic tumor antigens and consequently an enhanced response to immune checkpoint blockade. Even these studies highlight the fact that targeting nucleotide metabolism could be an interesting strategy to improve the immunotherapy response, additional exploration will be necessary to fully understand how nucleotides metabolism interferes with anti-tumor immunity especially because, in general, targeting nucleotides synthesis has immunosuppressive effects (Fig. 3).

## Ongoing clinical trials and future directions

While targeting metabolism theoretically offers various opportunities to improve the immune checkpoint blockade response or bypass resistance, at the moment, there is not yet a therapeutic schedule for the use of such therapies in standard care. However, several combinatory therapies are currently being or have been investigated.

The most advanced combinatory strategy for immunotherapy is probably the strategy that utilizes IDO1 inhibitors. As was already discussed, IDO1 is an enzyme responsible for tryptophan catabolism and conversion to kynurenine, which is a metabolite that will induce immunosuppression via the AhR signaling pathway. Studies showing that IDO1 is involved in the mechanism of resistance to various therapies, especially anti-CTLA4 treatment^102,103^, gave a proof of concept for the use of the combination. Numerous clinical trials are ongoing to test the efficiency of the combination of the IDO inhibitors Epacadostat, Indoximod, Navoximod, or BMS-986205 with anti-PD1, anti-CTLA4, or vaccine-based strategies^104,105^. However, even if the results of phase I and II clinical trials launched to test the efficiency of IDO1 inhibitors for the treatment of melanoma were encouraging^106^, the development of the drugs was stopped due to the lack of the efficiency of their combination with anti-PD1 for melanoma treatment^107^. The results for other cancer types will be particularly interesting as well as results of combinations with other drugs for determining the therapeutic potential of IDO1 targeting.

Concerning the amino acid arginine, inhibitors of arginase that block the myeloid cell-mediated inhibition of T cells are under investigation based on the in vivo result that showed that arginase blockade with CB-1158 synergizes with immune checkpoint blockade and adoptive T cell or NK cell therapies^108^. Unfortunately, the ongoing trial testing the triple combination of the arginase inhibitor INCB001158, the IDO inhibitor epacadostat, and anti-PD1 was stopped prematurely due to the negative result obtained from the combination of epacadostat and anti-PD1. Arginase plays a key role in immunosuppression in the tumor microenvironment, and it could be interesting to evaluate the efficiency of arginase inhibitors, not only in first-line treatment, but also in patients who have acquired resistance to immunotherapy, due to the importance of MDSCs in this phenomenon^109,110^.

Concerning the targeting of glutamine metabolism, there is one ongoing clinical trial testing the inhibition of glutaminase (GLS), the first enzyme involved in glutaminolysis, in combination with chemotherapy plus anti-PD1 for the treatment of non-small cell lung cancer. Furthermore, considering the impressive results obtained with the glutamine antagonist DON^27^, a clinical evaluation of the strategy appears to be essential.

Finally, the old anti-diabetic drug metformin has also found a second youth in trials testing its combination with immunotherapy. As we have seen previously in in vitro and in vivo data, targeting AMPK could be an interesting strategy to improve immune checkpoint inhibitor efficiency. Metformin is prescribed to more than 120 million people worldwide for type II diabetes mellitus. The extensive knowledge of its adverse events probably makes its combination with immunotherapy safer, and considering the numerous studies showing that patients treated with metformin have a low risk of cancer development, the results of the clinical trials will be useful (Table 1).

**Table 1:** List of currently ongoing clinical trials combining drugs targeting cell metabolism with immunotherapy strategies.

Metabolic target	Drug targeting metabolism	Immunotherapy strategy	Tumor type	ClinicalTrials reference
IDO1	BMS-986205	nivolumab (anti-PD1)/ipilimumab (anti-CTLA4)	Melanoma Non-Small Cell Lung Cancer	NCT02658890
	epacadostat	BN-Brachyury (MVA-BN-Brachyury based cancer vaccine)	Prostate Cancer	NCT03493945
	epacadostat	durvalumab (anti-PD-L1)	EBV^+^ Nasopharyngeal Cancer	NCT04231864
	INCB001158 plus epacadostat	pembrolizumab (anti-PD1)	Solid Tumors	NCT03361228
Arginase	INCB001158	pembrolizumab (anti-PD1)	Solid Tumors	NCT02903914
Glutaminase	telaglenastat (CB-839)	pembrolizumab (anti-PD1)	Non-Small Cell Lung Carcinoma	NCT04265534
AMPK	metformin	nivolumab (anti-PD1)	Non-Small Cell Lung Carcinoma	NCT03048500
	metformin	nivolumab (anti-PD1)	Colorectal Adenocarcinoma	NCT03800602
	metformin	sintilimab (anti-PD1)	Small Cell Lung Carcinoma	NCT03994744

Details on clinical trial design can be found on https://clinicaltrials.gov/.

## Conclusion

There is extensive evidence that targeting tumor cell metabolic adaptation can provide new therapeutic opportunities, especially when it is used in combination with immune checkpoint blockade strategies. The fact that PD-L1 expression in cancer cells has been demonstrated to stimulate glycolysis through AKT/mTOR activation perfectly illustrates the link that exists between the immune checkpoint and metabolism^14^. However, as we have seen before, cancer cells and immune effector cells, especially CD8 T cells, share various metabolic dependencies, which makes it difficult to target the metabolic adaptation of cancer cells without affecting tumor clearance induced by T cells. Thus, it is probably only by targeting essential pathways for cancer cells for which inhibition can be compensated for by immune cells that it will be possible to bypass the plateau we have reached with immunotherapies. An alternative strategy could be the use of combinations of approaches targeting cancer cell metabolism and adoptive T cell therapies. Indeed, the necessity of in vitro cell expansion provides an opportunity to genetically or pharmacologically compensate for T cell metabolic dependencies before patient transfer. In all cases, future work will be necessary to fully understand the metabolic dynamics within the tumor microenvironment, especially because the knowledge that we have accumulated via in vitro studies is hardly transferable in vivo or to patients due to the large excess of nutrients in culture media. Finally, trials to evaluate the potential of targeting the metabolic crosstalk between the tumor and the stroma should be designed more carefully and based on a deeper in vivo understanding if we wish to avoid a stinging failure like that of IDO inhibitors and be able to propose efficient combination strategies to bypass immune checkpoint inhibitor resistance.
